# Selections and Abstracts

**Published:** 1896-12

**Authors:** 


### SELECTIONS AND ABSTRACTS.

### The Discovery of Anesthesia.

The semi-centennial of anesthesia was observed by impressive exercises at the Massachusetts General Hospital, October 16th. A reception of the invited guests was held in the amphiteatre, in which, fifty years before, the first operation under ether was performed by Dr. J. C. Warren, the anesthetic being administered by the discoverer, Dr. W. T. G. Morton. An attempt was made to have this room present, as nearly as possible, the appearance it had half a century ago. Upon the walls were pictures of Dr. W. T. G. Morton, Dr. J. C. Warren, and other eminent physicians and surgeons of that day.

Dr. John Collins Warren, grandson of Dr. J. C. Warren, who did the first operation with ether, opened the exercises by reading a number of cablegrams showing the widespread interest in the celebration.

The chairman next introduced Dr. Robert T. Davis, of Fall River, who is one of the few surviving witnesses of the first application of ether in surgery. The operation, he said, was performed in the surgical amphitheatre of the Massachusetts General Hospital, by Dr. J. C. Warren, in the presence of a number of distinguished surgeons and physicians, among whom were Dr. Hayward, Dr. Jacob Bigelow, and his son Dr. Henry J. Bigelow. The Harvard medical class was also present. After some delay, Dr. W. T. G. Morton appeared with his apparatus, when Dr. Warren addressed the medical class, which had not been previously notified of the proposed experiment, stating, in substance, that there was a gentleman present who claimed that he had discovered that the inhalation of a certain fluid would produce insensibility to pain during surgical operations, with safety to the patient; that, as they were well aware, he had always regarded that condition as an important desideratum in surgery, and had permitted him to try the experiment. The patient was a young man who had had, since birth, a tumor under the jaw on the left side.

Dr. Warren had been applied to by Dr. Morton, a dentist, with the request that he would try the inhalation of a fluid which he said he had found to be effectual in preventing pain during operations on teeth. Dr. Warren having satisfied himself that the breathing of the fluid would be harmless, determined to use it on this patient. The apparatus consisted of a tube connected with a glass globe. This apparatus he then proceeded to apply, and after four or five minutes the patient appeared to be asleep. The operation consisted of an incision, about three inches in length, over the center of the tumor, through the skin and subcutaneous cellular tissue, and the removal of a layer of tissue. A curved needle was passed under and around the tumor and considerable compression made. To the surprise of Dr. Warren and the other gentlemen present, the patient did not shrink nor cry out, but during the insulation of the veins he began to move his limbs and to utter extraordinary expressions, and these movements seemed to indicate the existence of pain, but after he had recovered his faculties he said he had experienced none, but only the sensation like that of scraping the part with a blunt instrument, and he ever after continued to say that he did not feel any pain.

From that time forward it became the practice to employ it at the hospital in all operations of importance. Dr. Morton continued to administer it until it was proved that it could be easily and safely administered by others. The apparatus which he had used in the first and a few subsequent instances was soon abandoned as unnecessary, and attended with possible risk, and the concave sponge was substituted. Sulphuric ether as an anesthetic promptly passed into general use in Boston and throughout the State, and soon afterwards in public and private practice in the large cities of other States, followed by its employment all over the country wherever scientific surgery was practiced. Its fame crossed the ocean, and it rapidly became a necessary adjunct to surgery in Europe as well as here, and beyond, even to the utmost limits of civilization—it did not stop there, but among savage tribes and barbarous races in distant continents and islands it followed the footsteps of the explorer, the trader and the missionary on its divine errand of mercy to mankind. It is impossible to estimate or comprehend the importance of this beneficent discovery.

Dr. John Ashhurst, of Philadelphia, read a paper on "Surgery Before the Days of Anesthesia."

A study of the condition of operative surgery before the days of anesthesia revealed, on the one hand, a picture of heroic boldness and masterly self-control on the part of the surgeon, and on the other a ghastly panorama, sometimes of stoic fortitude and endurance, sometimes of abject terror and humiliation, but always of agonizing wretchedness and pain on the part of the unhapyy victim, man or woman, whose necessities required a recourse to the surgeon's aid. And from our vantage ground of half a century's experience, it was difficult for us to understand why, with the constant and persistent efforts made by surgeons in past ages to lessen the pain of operations, and with the gradual but continuous accumulation of facts, showing that by certain agents pain could be temporarily abolished without danger, the eyes of all patients as well as practitioners yet seemed to be holden; and why, science and art working with a common object, if independently, though the whole world seemed to be trembling on the verge of the discovery, it yet was not until fifty years ago to-day that the crucial experiment was made in this hospital, and that surgical anesthesia became a glorious reality.

It was not an unheard of thing for the surgeon's presence of mind to fail him in difficult operations at the present day, but at least the patient, unconscious through the blessing of anesthesia, did not know it. Celsus urged pitilessness as an element of success in operating. Sir James Simpson, of Edinborough, shortly after beginning his professional life, was so affected by seeing the agony of a woman whose breast was amputated, that he resolved to abandon the profession; but later reconsidered this, and asked: "Can nothing be done to make operations less painful?" Twenty years later he introduced chloroform. After Admiral Nelson's elbow was shattered, he underwent amputation with the same firmness and courage that always marked his character, and yet so painfully was he affected by the coldness of the operator's knife, that he gave a standing order to his officers that hot water should always be at hand, so that the unfortunate victim might at least have the poor comfort of being cut with warm instruments.

Case after case was narrated by Monfalcon in which men submitted themselves with the utmost calmness and fortitude to the hands of skillful operators instantly falling into collapse after the first incision, and without undue loss of blood quickly succumbing to the depressing effects of simple shock and pain. Opium and alcohol were the only agents that continued to be regarded as of practical value in diminishing the pain of operation, though the disadvantages of their employment were, of course, recognized. Alcohol, pushed to the point of intoxication, had been used by some surgeons; but yet surgeons went on cutting and burning, and patients went on writhing and screaming, until on the 16th day of October, 1846, in the Massachusetts General Hospital, Dr. John C. Warren painlessly removed a tumor from a man who had previously been etherized by Dr. William T. G. Morton, and surgical anesthesia became the priceless heritage of the civilized world.

Dr. David W. Cheever, Professor Emeritus of Surgery in Harvard University, presented the next paper on "What Anesthesia Has Done for Surgery."

The reader said there was no more universal instinct than to dread the knife, to shrink from an operation, to fear pain. All this had been annulled by anesthesia. The shock of the operation was largely diminished. Anticipation was done away with; pain was prevented; shock was reduced. The patient consented to operation earlier. He did not wait till life became unbearable, but calmly contemplated surgery as the natural and easy channel of relief; hence, his chances of benefit from an operation were much increased. So much was done for the patient.

To the surgeon anesthesia gave the patient a sleep, motionless, senseless. He need not hurry, he need not sympathize, he need not worry, he could calmly dissect as on a dead body; heedful only that the etherizer was competent, that the breathing and pulse were watched, that the operation was not prolonged beyond the verge of exhaustion. The surgeon, then could do better work, could be more careful, could pause and consider, could choose his steps, could be deliberate, if not dextrous. He could even summon the aid of the pathologist and his microscope, who in ten minutes, while the patient slept, could decide the nature, the innocence or malignacy of the tumor he was removing. It was not too much to say that all the finer work of plastic and abdominal surgery dated from the discovery of anesthesia. It could not have been done before; neither surgeon could persist nor patient endure. Perhaps one thousand ovariotomies were done by Sir Spencer Wells before antiseptics were much practiced, but all since anesthesia was known. Formerly the surgeon was esteemed for dexterity; now he was esteemed the great surgeon who, to judgment, adds dexterity, and to dexterity patience. Anesthesia was the necessary precursor of asepsis. Without the former the latter could not be what it now is. Even if antiseptic agents were used in dressing wounds, the operations which caused the wounds could not have been done aseptically without anesthesia. The essence of asepsis was detail, tedious rules and precautions, prolonged and accurate dressings. All this required time, immobility, unconsciousness. The surgeon could not stitch the most delicate tissues with accuracy, match the bowel or bladder, so that it would not leak, on a conscious and quivering patient. Anesthesia and asepsis must be inseparable. Visceral surgery, which dealt with great serous cavities, and which constituted the proud distinction of modern surgery, depended on anesthesia first, and on asepsis afterwards.

Dr. J. P. Reynolds, formerly Professor of Obstretrics in Harvard University, spoke of "The Relation of Anesthesia and Obstetrics."

Dr. Reynolds said that in operative obstetrics, in the high and difficult application of instruments, in the introduction of the hand for version and extraction, anesthesia produced the same admirable results as in other surgical proceedures. It brought unconsciousness to the patient and afforded the operator ample time for exact diagnosis and opportunity to work with accuracy and delicacy. Anesthesia in the induction of premature labor was of great assistance. The service of ether in puerperal convulsions was still more striking; it had no power to cure, but we gained an opportunity for treatment—the emptying of the uterus. In normal labor due anesthesia was free from every shadow of danger. The anesthetic should be allowed only during the uterine contraction, the time of positive pain. In this manner ether might be continued from the beginning of labor to its close. Whenever the attendant saw that the woman's endurance of pain began to tell upon her patience and courage the moment for ether had come. It should be kept up as long as this need lasted, no longer. In its first employment a lull often came in the uterine action, the patient sinking into a much needed sleep, to which soon succeeded a more vigorous advance. In the earliest pains some observers held that chloral in suitable doses gave better sesults.

"The Influence of Anesthesia upon Medical Science" was able discussed by Dr. W. H. Welch, of Johns Hopkins University.

The discovery of ether, the reader said, was a triumph for the experimental method. It was important to emphasize that the utility of the discovery of anesthetics was not limited to their application to surgical, medical and obstetrical art, but that this discovery had been of great service in experimental medicine. The introduction of anesthesia came at a period of awakened activity of investigation. Dr. Welch discussed, at some length, the relation of experiments upon animals to the advancement of medical knowledge. He maintained that in all exepriments upon animals insensibility to pain was first secured in all cases in which this condition would not defeat the object of the experiment.

The last paper was by Dr. Charles McBurney of New York, on "The Surgery of the Future."

Without anesthesia the best of modern work in surgery would have been impossible. Through the discovery of asepsis and anesthesia, surgery had become a gentle art. It would almost seem as if the future would hold for us no obstacles, such had been the advance in the past. In the immediate future the greatest surgical victories were to be won by bacteriology. Through it diphtheria and tetanus had been brought within the list of frequently curable diseases. Would not the discovery soon be made by which we should be able to destroy the sepsis-producing organisms, no matter how wide its distribution. Operative surgery had done much to overcome the terror of cancer. The discovery of the true nature of the disease, whether it owed its existence to a living organism, was for the future. Bacteriology was and ever would be in the hands of most brilliant men, and surgery was destined to attain many of its most brilliant triumphs through the aid of this army of valuable co-workers. Through the study of the blood we might expect in the future an exact and early diagnosis in many surgical disorders which now were appreciated in a stage too late for beneficial treatment. The operating surgeon of the future would know when to interfere and when to withhold his hand; he would know, better than we, who were actually moribund. He would not be tempted by the plea that the patient must die and should be operated on as a last resort.

He would not attempt to cure with the knife the little microcephalic child. He would have at his disposal the large experience of the surgeons of to-day. We might confidently look forward to improved diagnosis of surgical disease. The future surgeon would enjoy a much closer and more intimate relation with his brother, the physician, than had ever existed between them before, for what could be cured by surgery only would be far better appreciated by both surgeon and physician than at present. Above all, the surgery of the future would attract to its enthusiastic study and practice finer and finer men, in whose hands we might safely leave the development of our science. A single glance at the faces of the students who collected in the operating rooms would show what a change had occurred in the last twenty-five years. For this too we must ever be grateful to anesthesia, which, in removing the torture of surgery, had robbed it of what repelled many sensitive natures; and the science of asepsis, by rendering complete success in surgical work possible, would excite the most devoted enthusiasm from many scientific men, who soon would have become sick at heart over the failures of former times. Through the vast experience of the recent past, the surgeon of the future would find many of the coarser problems already solved, and those that remained would stimulate him by their difficulty. He would be an accomplished anatomist and a physiologist; he would study medicine and pathology first, and then general surgery. If he specialized his practice he would do so only after a large general experience, and he would borrow from every science all that could contribute to the perfecting of his work.

A beautiful poem, entitled "The Birth and Death of Pain," was then read by Professor S. Weir Mitchell, of Philadelphia.—*International Journal of Surgery.*

---

### SENILE ENDOMETRITIS AND VAGINITIS.

Dr. Augustin H. Goelet, Professor of Gynecology in the New York School of Clinical Medicine, presented a paper on this subject at the October meeting of the New York Medico-Surgical Society (*Medical Record*). He thinks if the general practitioner will consider that women past the menopause may be liable to chronic inflammation of the endometrium, and will look for symptoms denoting it, he will find that it is by no means infrequent, and that many obscure troubles in women at this age will be cleared up and their suffering relieved.

The atrophic changes which occur at this time, and which are due to a diminished pelvis circulation and impaired local nutrition, are directly responsible for this condition. He agrees with Skene that this process, in many cases, is a degeneration rather than an inflammation. During the first year or two after the monopause, however, it undoubtedly exists as a chronic inflammation, but contraction and narrowing of the canal of the cervix causes retention of the secretion, which becomes acrid and destroys the mucous membrane.

The general mal-nutrition which accompanies this condition is regarded more as a result than a cause of the disease, and disappears upon the establishment of drainage and improvement of the local condition. The disease is characterized by a purulent or muco-purulent, discharge which is at times very acrid and irritates the vagina and vulva. The rugæ are effaced, and the surface of the vagina is smooth and glistening in places, with here and there minute ecchimosed papillæ.

The same method of treatment which would be applicable for endometritis in younger women would not be suitable in this condition. It is true that drainage and absolute cleanliness of the uterine cavity are essential, but this cannot be accomplished by dilatation in the usual manner with curettage. The mucous membrane in advanced cases is already destroyed, and the closure of the canal of the cervix from bands of cicatricial tissue would make ordinary dilatation dangerous, since rupture would result. The author points out that this may be accomplished safely by means of conical electrodes employed with the negative pole of the galvanic current, and cautions that only a moderate strength of current should be employed, and cauterization should be avoided. When dilation has been thus accomplished, a special, small, double current irrigator is inserted, and the cavity is washed out with a weak solution of lysol. This irrigator can be utilized as an electrode, and the current is turned on while the irrigation is going on, thus producing a moderate stimulation of the surface of the endometrium through the medium of the irrigating fluid. After cleansing the surface of the vagina and vulva, it is dusted with a bland, non-irritating antiseptic powder, markasol, and a solution of the same is used as a vaginal douche once or twice a day, as required. For vesical tenesmus the urethra is dilated with the same electrodes, and the bladder is washed out with a one per cent. solution of markasol. The same solution is used for the rectum.

Cicatricial contraction of the vagina in advanced cases may prevent the use of a pessary to correct a misplacement if this complicates the case. In these cases, as well as where the organ is prolapsed, the author would prefer ventral suspension to hysterectomy.

Senile endometritis is usually regarded as most intractable, and is trying for both physician and patient. Yet the author says he knows of no gynecological disorder, the treatment of which is undertaken with more certainty of success.

---

### ARSENIC IN THE TREATMENT OF GASTRALGIA.

In the *Lancet* for July 4th, Sir James Sawyer, M.D., of Birmingham, says: "Further observation in practice has confirmed my favorable opinion of the curative efficacy of arsenic in the various painful neuroses included under the name gastralgia. I have already laid before the profession my earlier experience in the subject. Romberg's well-known description of gastralgia is classical. He distinguished two forms of the malady—gastrodynia neuralgica, which he held to be hyperæsthesia of the gastric branches of the pneumogastric nerve, and neuralgia cœliaca, which he attributed to hyperæsthesia of the solar plexus. Clinical experience confirms the views of Niemeyer and of Henoch that this distinction is difficult and of doubtful utility in practice. Gastralgic affections, severe and slight, are rare in hospital practice, and frequent among private patients, especially in those of nervous temperament. I need scarcely observe that for obvious reasons the diagnosis of gastralgia is one which should neither be lightly made nor negligently maintained. But pain arising in the stomach when the organ is empty and relieved by the ingestion of food is almost diagnostic, as the late Dr. Wilson Fox taught, of its nervous nature and origin. With due regard to the causal concomitants and antecedents of gastralgia, arsenic cures the disease. It is best to give the drug in a pilular form. I exhibit a twenty-fourth of a grain of arsenious acid made into a pill with two or three grains of some tonic vegetable extract, such as gentian, three times daily halfway between meals. Scarcely any other medicinal treatment is needed in cases of moderate severity, and the use of the remedy should be continued for some weeks. In severer cases I use counter-irritation to the epigastrium or duly proportioned activity. I have usually found a full and varied dietary suit gastralgic patients far better than a restricted 'dyspeptic' regimen. It is in such cases that Trousseau's maxim is so true—that we should know what a patient does eat before we advise him upon what he may feed."

---

### THE TREATMENT OF OTORRHEA.

In a late paper on this subject, Dr. E. B. Dench, of New York, said that a profuse chronic purulent discharge from the ear, which had lasted for more than two months, evidently came from the middle ear, and of course the membrana tympani would be found perforated. If the purulent discharge was scanty, it was probable that its source was the external meatus. If the discharge was serous and not very abundant, it probably came from the middle ear. While pressure in front of the tragus or traction on the auricle in the adult would elicit pain in cases of disease in the external auditory canal, this sign did not hold true in children, because in them the bony canal was absent. Where the drainage was free, the discharge would cease as soon as all necrotic tissue had been discharged. The first object in treatment should be to maintain efficient drainage, and then keep the parts aseptic. The speaker preferred syringing out the ear to the use of gauze drainage. The process of syringing could be done thoroughly only when the precaution was taken to draw the auricle outward, upward, and backward in the adult, and outward, downward, and backward in the child. Peroxide of hydrogen should not be instilled into the ear in cases of purulent otorrhea, because the solution was irritating and the quantity of gas liberated when it came in contact with pus was apt to cause an injurious degree of pressure within the ear. The best solution for irrigating the ear was a warm 1-to-3,000 or 1-to-5,000 solution of bichloride of mercury. The patient should be cautioned against plugging the ear with cotton in the interval between the irrigations, as this practice was very apt to result in the formation of feruncles about the meatus. All forms of powders were objectionable in the ear, as they formed hard crusts with the discharge, thus obstructing drainage and irritating the ear. In conclusion, Dr. Dench quoted statistics, showing that out of 820 cases of suppurative disease of the middle ear, 2.5 per cent. had terminated fatally.—*N. Y. Med. Journal.*

---

### TREATMENT FOR THE FALLING OUT OF HAIR.

*Extraits du Formulaire Clinique de Vienne.* Translated by Rutherford Gradwohl:

| | | |
|---|---|---|
| ℞ | Tinturæ cinchonæ rubræ.................................... | ℥ j |
| | Tincturæ cantharidis........................................... | m. xxx |
| | Acidi Phenici.................................................... | gr. xxx |
| | Tincturæ ignatii.................................................. | m. vijss |
| | Aquæ cologne, | |
| | Oleum coco, aa, q. s. ad...................................... | ℥ iv |

M. Sig.—Make one or two applications daily.

Or wash with the following exciting mixture:

| | | |
|---|---|---|
| ℞ | Tinc. cantharidis | gr. v |
| | Essence rosemarini, | |
| | Essence lavender, aa | gtt. x |
| | Eau de Cologne | ℥ iij |
| | Olei sabinæ | grs. xv |
| | Spts. vini | ℥ j |

Or,

| | | |
|---|---|---|
| ℞ | Liquid vaseline | ʒ jss |
| | Pilocarpine | gr. ss |

Or, finally, apply:

| | | |
|---|---|---|
| ℞ | Quininæ sulphatis | gr. xv |
| | Acidi aceticin, | |
| | Acidi phenici, aa | gr. v |
| | Misturæ oleo-balsamic | ʒ iijss |
| | Olei ricini | ʒ jss |
| | Glycerini | ʒ ss |

—*Ex.*

---

### A CODE OF MEDICAL ETHICS.

To the graduating class in the medical department of the University of Pennsylvania, Dr. H. C. Wood said: "Consider every member of the profession as one of your own family, and having an inherent right to your medical services, but do not abuse this right; consider any discovery or invention you may make as belonging to the general profession; never in any way laud your own medical skill or attempt to supplant in public or private estimation one of your medical brethren; join as soon as may be the incorporated companies of your fellows for scientific and social intercourse, and for the cultivation of that professional conscience which often binds men more closely than their personal sense of right and wrong; through good and ill report, stand by members of your own profession, unless they be guilty of moral evil."—*Medical Age.*

---

### STERILIZATION OF SYRINGES.

Hofmeister has found that leather, like catgut, can be effectively sterilized without the slighest injury, by soaking it in a 2 to 4 per cent. solution of formalin and then boiling it. Syringes that screw together instead of being glued or cemented, are better adapted to this method. The water in which they are placed to boil must not be hot enough to crack the glass.—*Cbl. f. Chir.*, July 4,

